# Nutritional Status of Elderly Patients after Coronary Artery Bypass Surgery

**DOI:** 10.3390/ijerph16020226

**Published:** 2019-01-15

**Authors:** Lucyna Ścisło, Aleksandra Bizoń, Elżbieta Walewska, Magdalena Staszkiewicz, Radosław Pach, Antoni Szczepanik

**Affiliations:** 1 Department of Clinical Nursing, Faculty of Health Sciences, Jagiellonian University, Medical College, ul. Kopernika 25, 31-501 Krakow, Poland; bizon.aleksandra@gmail.com (A.B.); elwalewska@gmail.com (E.W.); baranmagdalenaa@gmail.com (M.S.); 2 Department of General, Oncological and Gastroenterological Surgery, Jagiellonian University, Medical College, ul.Kopernika 40, 31-00 Krakow, Poland; radoslaw.pach@uj.edu.pl (R.P.); antoni.szczepanik@uj.edu.pl (A.S.)

**Keywords:** coronary artery bypass, malnutrition, overweight, obesity

## Abstract

Surgical trauma can result in immobilization of biological material, degradation of muscle proteins, synthesis of acute-phase proteins in the liver, occurrence of catabolism phase and anabolism simultaneously, and as a consequence weight loss and nutritional deficiencies. The aim of this study was to assess the nutritional status of patients with ischemic heart disease subjected to coronary artery bypass surgery and physical activity and postoperative complications. The analysis among 96 men included total number of lymphocytes (TNL), body mass index (BMI), case history of a patient and results of laboratory tests. The activities of daily living (ADL) and the mini nutritional assessment (MNA) questionnaires were used. According to TNL, before the procedure malnutrition occurred in 46% of patients. BMI revealed overweight in 62.5% and obesity in 26.0%. After the surgery, no changes were observed. According to MNA, 59% of patients before the surgery were at risk of malnutrition. After the operation, the number of people at risk of malnutrition increased by 50% (*p* < 0.0001). The correlation was noted between BMI and patients’ efficiency in the fifth day after the surgery (*p* = 0.0031). Complications after the surgery occurred in 35.4% of patients. After the surgery, the risk of malnutrition increased, decreased activity and complications occurred more frequently in people with underweight, obesity, and overweight than in people with normal BMI.

## 1. Introduction

Surgical procedures performed in the elderly are part of daily clinical practice [1]. Surgical trauma can result in immobilization of biological material, degradation of muscle proteins, synthesis of acute-phase proteins in the liver, occurrence of catabolism phase and anabolism simultaneously, and as a consequence weight loss and nutritional deficiencies. Low levels of albumin lead to edema and mucosal atrophy as well as excessive bacterial growth which, in turns disrupts absorption. Deficiency of immunoglobulin A (IgA), vitamin C, and glutamine favors the occurrence of immunological disorders. A decrease in weight of over 4.5 kg within three months increases the risk of perioperative mortality 19 times [2,3,4]. Frequently, prior to surgery, malnutrition is unrecognized and untreated. In hospital, the poor nutritional status often deteriorates, thus extending the time of hospitalization and increasing the risk of complications and death. It stands to reason that the cost of treatment also rises. In hospital, declining malnutrition is often a metabolic consequence of the disease, lack of food intake during the perioperative period but also the result of perioperative injury. It can worsen a patient’s prognosis after leaving the hospital and in surgical patients it often contributes to impaired cellular and humoral immunity, both of them being responsible for the increased incidence of infection, such as pneumonia and sepsis. This can be accompanied by compromised wound healing and wound dehiscence. Other complications are also observed, such as fistulas, pressure ulcers, even an increased risk of bone laceration and fractures. Malnutrition characterized by a reduced level of proteins, including albumin, prealbumin, transport proteins, and transferrin often leads to muscle wasting, a decline in the patient’s immunity, and disorders of internal organs functioning. Weakening of muscle strength is often concomitant with a decline in psychomotor performance. There are disturbances of water and electrolyte metabolism and deficiency anemia. Weakened intestinal motility is associated with impaired absorption and digestion, as well as increased permeability of the intestinal barrier. Malnutrition can also trigger cardiovascular, respiratory, and urinary system disorders. Steatosis of liver cells occurs, which is associated with decreased production of proteins. Pancreas mass decreases and digestive enzyme secretion is disturbed [2,3,4].

Frequently, before surgery, malnutrition of patients is undiagnosed and untreated, then increases in the hospital prolonging hospitalization, adding to the risk of complications and death, and also increasing the cost of treatment. Malnutrition deteriorating in the hospital is often a metabolic consequence of the disease, inadequate food intake during the perioperative period as well as the result of perioperative injury. All of these worsen patients’ prognosis after leaving the hospital. In surgical patients, it often leads to impairment of cellular and humoral immunity, and among other things, is responsible for the increase in the incidence of infections and difficulties in wound healing process [2,3,4].

Improper nutritional status is characterized not only by malnutrition, but also by overweight and obesity. Obesity is currently a social problem and the cause of metabolic disorders. It creates a number of problems in everyday life; difficulties with movement, constraints in private life and professional life activities, lack of acceptance by the environment [5].

The number of diseases accompanying obesity includes: type II diabetes, hypertension, lipid disorders, atherosclerosis. Excess visceral fat promotes the development of chronic, systemic inflammation which is associated with abnormal production of cytokines and activation of proinflammatory signals [6,7,8,9].

Elderly people are particularly vulnerable to the occurrence of nutritional disorders. They are affected by general factors, but also age-specific, biological and psychosocial ones. The most significant are: process of aging of specific organs, loss of appetite, disturbances of taste and smell, systemic diseases, pharmacotherapy, limited activity level, material status and mental health [10]. In the case of elderly people, eating disorders concern mainly patients with various types of disorders of respective systems. One of the most common disorders is atherosclerosis, which is associated with the occurrence of negative cardiovascular events and often subject to surgical treatments [10]. 

In patients with myocardial ischemia, percutaneous coronary interventions (PCI) or coronary artery bypass graftings (CABG) are performed [11,12,13,14,15,16].

Patients after CABG are exposed to secondary obstruction of operated vessels, cardiac disorders, surgical site infection, cardiorespiratory, multi-organ failure, and neurological disorders (stroke, disturbances of consciousness). Cognitive function in terms of non-verbal long-term memory, attention, concentration, psychomotor speed and flexibility may also deteriorate. People qualified for surgery are not only getting older, but they also have more and more comorbidities, and this affects the occurrence of neuropsychological disorders after the procedure. Operative trauma also leads to a decrease in the immunity, and consequently to nutritional disruptions and an increased risk of the inflammatory process initiation [11,12,13,14,15,16].

An important aspect in patients’ care after CABG is early diagnosis of malnutritional disorders. It helps to properly prepare the patient for surgery and reduces the risk of postoperative complications [17].

For the purpose of early detection of malnutrition and the associated immunodeficiency, an analysis of the total number of lymphocytes (TNL) is performed. A commonly used parameter for measuring the nutritional status is the body mass index (BMI). Malnutrition is always associated with a decline in immunity and serves as a screening tool. When interpreting TNL it should be remembered that in addition to the nutritional status, stress, acute diseases, infections and cancer also affect its value. In clinical practice, scales and questionnaires are also used to assess the nutritional status of patients, including the mini nutritional assessment (MNA), the Malnutrition Universal Screening Tool (MUST), the Subjective Global Assessment (SGA), and for the assessment of the risk of abnormal nutrition—the Nutritional Risk Screening 2002 (NRS 2002) [18,19,20,21].

## 2. Materials and Methods

Evaluation of nutritional status should be mandatory and performed during admission to hospital. It seems necessary to implement nutritional treatment before surgery and in the first days after surgery in patients at risk of malnutrition. Furthermore, it seems inevitable to study the impact of nutritional treatment on further outcome and hospitalization of a patient and to plan a balanced diet tailored to the patient’s needs.

The aim of the study was to assess the nutritional status of patients with ischemic heart disease before and after coronary artery bypass surgery and to determine the correlation between nutritional status and physical activity and postoperative complications.

The study was conducted between February and June 2016 among 96 men hospitalized in the department of cardiovascular diseases at the hospital in Poland.

The applied inclusion criteria were male gender, age 65–79, planned admission for the first aortal-coronary bypass surgery and at least five-day hospitalization. Exclusion criteria were female gender, age above or under 65–79, consecutive surgery, sudden admission procedure and hospitalization shorter than five days. The group of patients did not receive any nutritional treatment. Since the first postoperative surgery, patients received a diet indicated for patients with cardiovascular burden.

The analysis includes the results of an immunological test-TNL (Total Number of Lymphocytes), index BMI (Body Mass Index), types of postoperative complications. The Katza scale—activities of daily living (ADL), and the Minimal Nutritional Assessment (MNA) questionnaire were used. Measurements were carried out by the authors of the study. In order to obtain TNL results, a sample of venous blood was taken from the patients and put into a test tube (2.6 ml) with EDTA content to determine full blood cell counts with a blood smear. The test was performed by fluorescence flow cytometry.

TNL is percentage ratio of lymphocytes and the number of leukocytes, divided by 100. The normal level of TNL in the peripheral blood is >1500 in 1 mm^3^ of venous blood. Mild malnutrition is diagnosed when TNL is 1200–1499, moderate malnutrition when TNL is 800–1199 and severe malnutrition occurs when TNL <800 [22].

On the day the patient was admitted to the hospital, the height and weight were measured. The BMI index was calculated by dividing the subject’s body mass expressed in kilograms by the square of his/her height expressed in meters. Normal BMI range is 18.5–24.9 kg/m^2^. Values below 18.5 kg/m^2^ were considered to indicate malnutrition, over 25.0–29.9 kg/m^2^ overweight and over 30.0 kg/m^2^ obesity class I, II, or III [23].

On the first day of hospitalization, a nutritional interview with the patient was carried out using the MNA scale. The proper nutritional status is declared within the threshold of 24–30 points. 17.5–24 points indicate a threat of malnutrition, while <17 points is diagnosed as malnutrition [24].

Also, immediately after the admission of the patient to the hospital, the degree of independent functioning in everyday life was determined on the basis of the ADL scale.

The maximum number of points to be scored is 6. The lower score refers to the total or partial inability of the patient to function independently and the need for help [25].

Evaluation of the nutritional status of patients, and the level of their efficiency was determined before the surgery, on the day of admission to the hospital and in the fifth day after the surgery. Additionally, on the first day after the procedure, the TNL level was assessed.

Based on medical records, the analysis of postoperative complications was made using patients’ medical history. They included cardiovascular, respiratory, urinary, neurological, and surgical disorders (prolonged drainage of bloody discharge, infection of the surgical wound, instability of the sternum).

Each patient gave consent to conduct tests and to process personal data. Patients were ensured about anonymity of the study. Ethical principles of applying tests were observed in accordance with the principles of the Helsinki Declaration.

The study used descriptive statistics methods and a qualitative analysis. 

Analyses of variables were made using χ2, Mann–Whitney and Kruskal–Wallis tests. The correlation between the variables was checked using statistical tests taking the significance level for *p* < 0.05. The calculations were made in the SPSS program.

## 3. Results

Among 96 subjects were men aged 65–81. The average age was 69 years (SD = 5.36).

### 3.1. TNL Characteristics

The average TNL value in the subjects was 1862.59 (SD = 2383.78). Nearly half of the patients (54%) had TNL values above 1500, indicating proper nutritional status of these patients. Based on these results, 39% of patients were characterized as having mild malnutrition and 7% as having moderate malnutrition. 46% of patients with TNL values indicating malnutrition had a higher risk of postoperative complications related to the weakening of the immune system. The mean TNL value on the first day after CABG was 1566.62 (SD = 550.9). In 47% of patients this value was >1500 indicating proper nutritional status of these patients. A mild malnutrition was found in 37.5% of the subjects, while the moderate in 15.6%. The majority of the patients (53.1%) were malnourished. The day after surgery showed the average TNL value of 1544.83 (SD = 311.43). In 51% of the patients, nutritional status was within norm. A mild malnutrition was noted in 40.6%, whereas moderate malnutrition was revealed in 8.3% of patients. On the fifth day, the number of people with proper nutritional status and mild malnutrition increased compared to the first day, and the number of people with moderate malnutrition decreased. The number of people with proper nutritional status and mild or moderate levels was similar to the period before CABG. There was no statistically significant relationship between TNL values before and after surgery (*p* > 0.05) (Table 1 and Table 2).

### 3.2. BMI Characteristics

The mean value of BMI in the patients qualified for CABG surgery was 27.92 kg/m^2^ (SD = 2.65). In 2% of the patients, underweight was found, in 63% of the patients overweight, and 26% were found obese. Correct body mass was observed in only 9% of the patients. 88.5% of the subjects had BMI above the recommended norm. On the fifth day, the mean BMI value decreased slightly and amounted to 27.52 (SD = 2.66). The number of people with correct body weight increased to 11% and those with overweight to (70%), reduced the percentage of people with obesity (17%). There was no statistical relationship between BMI values in the two study stages (*p* > 0.05) (Table 3 and Table 4).

### 3.3. Characteristics of MNA

The mean value of MNA for the patients prepared for CABG was 22.89 points (SD = 1.53). According to MNA, 40% of the patients had proper nutritional status, 59% were at risk of malnutrition and 1% had malnutrition. On the fifth day after surgery, proper nutritional status was found in 5.2% of the patients. The number of the patients at the risk of malnutrition also increased (92.7%) compared to the pre-operative period (59.4%). The occurrence of malnutrition was reported in 2.1% of the patients. A statistically significant correlation was found between MNA values before surgery and MNA values on the fifth day after surgery (*p* < 0.0001) (Table 5 and Table 6).

### 3.4. ADL Characteristics

Thanks to ADL analysis, the level of the patients’ functioning was determined. Before the procedure, 81.3% of the patients were fully functional. The subjects with impaired functional status constituted 18.7%. On the fifth day after surgery, no significantly functionally impaired patients were observed, while only 33.3% of the patients were fully functional. This amounts to 48% fewer cases than before the procedure. It was observed that specific daily living activities were significantly related to the perioperative period (*p* < 0.0001), except for self-feeding (*p* = 0.3666). It was demonstrated that the extent of coronary artery bypass surgery negatively affects functional status within basic activities (*p* < 0.05) (Table 7).

Analyzing TNL before the procedure, no significant relationship was shown between the patients’ state of nutrition and their functional status. However, based on TNL, there was a significant relationship between the nutritional status of the patients on the fifth postoperative day and their functioning. It was noted that the patients with impaired functioning more often (64.1%) than these with proper functional status (18.8%) were malnourished (*p* < 0.0001) (Table 2 and Table 8).

The study showed a statistically significant relationship between BMI and the functional status of the patients (*p* < 0.0001). Only the patients who had BMI within the norm or BMI indicating underweight were fully functional. Overweight subjects were also more likely to be fully functional. On the other hand, impaired functional status dominated among obese patients. Statistically, BMI value significantly influenced functional status of the patients on the fifth day after CABG (*p* = 0.0031). Functionally effective patients more often had BMI within the norm (28.1%), while people with impaired functional status showed BMI above the norm (93.8%) (Table 9).

When analyzing MNA, no significant correlations were found between the patients’ nutritional status and level of their self-functioning (*p* > 0.05) (Table 10).

### 3.5. Postoperative Complications

It was found that postoperative complications occurred in 35.4% of subjects. The most frequent cases were sternum instability (16.7%) and cardiovascular disorders (14.6%). Within the small group of subjects, neurological disorders, prolonged bloody discharge, or postoperative wound infection (4.1%) occurred. There were no complications from the respiratory and urinary systems in the study group.

Due to the highest number of disturbances reported in the nutritional state based on BMI, the correlation between BMI and the occurrence of postoperative complications in the patients was assessed. A significant correlation was found between BMI and the occurrence of postoperative complications in the patients (*p* = 0.0351). The analysis showed that postoperative complications occurred in all underweight patients, 56.3% of the overweight group and 31.3% of the obesity. Only 18.2% of subjects with normal BMI had complications.

## 4. Discussion

Nutritional status is an indicator conditioning proper recovery of the patient after the surgery. Early identification of the abnormal state of nutrition and the implemented measures reduce the risk of post-operative complications resulting from malnutrition or excessive body weight. Caring for the geriatric patient after an operation should combine not only the risk associated with the surgical trauma, but also changes characteristic of the patient’s age. Consequences resulting from the aging process contribute to the occurrence of biological, mental, and social disorders. As a result, elderly people are more at risk of complications after surgery, including complications resulting from abnormal nutritional status [26].

Over the past 25 years, the number of patients over 75 years of age who have undergone surgery has increased. Older age is a predictor of an increased risk of postoperative complications, prolonged hospitalization and mortality. Italian researchers indicate that it is necessary to pre-assess the nutritional status of patients due to the relationship between nutritional status and high risk of postoperative complication [27].

Our own studies based on BMI, TNL, and MNA determined nutritional status of patients after CABG procedures. Most of the subjects based on BMI (n = 65, 62.5%) were overweight and 25 patients (26%) had obesity. The results of other authors also showed that the majority of patients with cardiovascular disease had excessive body weight. The average BMI of men was 30.29 kg/m^2^ [28]. The reasons were poor eating habits. This shows the need for constant demand for education regarding correct nutritional habits. Luisi ML et al. point to the need for individual nutritional counseling for people with cardiovascular disease, especially for those with a poor nutritional status [29].

In our own study, patients’ functional status was also determined based on ADL level. It was shown that people with BMI indicative of obesity were less well-functioning than people with BMI within the norm. The majority of people (66.7%) after surgery on the fifth day had impaired functioning, the others were fully functional (33.3%). People with malnutrition characteristics defined on the basis of TNL showed reduced functional effectiveness. Polish researchers show that patients’ complete activation should occur on the fifth to seventh day after CABG, while between two and six months after the operation, the effectiveness in the area of independent functioning should be significantly improved [30,31,32]. 

Furthermore, it was proved based on TNL that nearly 40% of patients were malnourished both before and after the procedure. There was no significant difference in TNL values between the study periods. On the basis of MNA on the fifth day after CABG, the number of the patients at the risk of malnutrition increased compared to the period before the procedure. This may confirm the impact of surgical trauma on metabolism and nutrition [33]. MNA seems to be a reliable tool to assess the risk of malnutrition before and after treatment among elderly people [34].

Complications after CABG were more often present in the patients with overweight or obesity. In addition to complications that occurred in the studied patients, others also report respiratory complications, wound infections and thromboembolism [35]. Obesity is also a predictor of premature death in patients after cardiac surgery due to a sternum infection or renal failure [36]. Lopez-Delgado J.C. et al. observed frequent septic and cardiac complications in obese patients undergoing surgical procedures [37]. Excessive body weight is also the cause of postoperative atrial fibrillation [38].

Malnutrition is a serious problem for patients who are scheduled to undergo cardiac surgery. Moreover, it also affects the condition of the cardiovascular system. Researchers dealing with malnourished children all over the world showed that the degree of malnutrition is correlated with the decrease in myocardial mass and a number of complications associated with it [17]. It was also revealed that malnutrition concurrent with coronary disease has a very negative effect on patients with other chronic diseases, including renal failure. Malnutrition and atherosclerosis are one of the causes of increased mortality in chronic kidney disease (CKD) [39]. They are also predictors of increased mortality among hemodialyzed patients. The mortality rate in these patients with mild to moderate malnutrition was 37% and 67% with severe malnutrition. The necessity of hospitalization was found in 43 (46%) patients and was much more frequent in malnourished patients compared to properly nourished (77% vs. 32%) [40].

The risk factor that increases morbidity and post-operative mortality is pre-operative malnutrition [41,42,43]. Correlations between malnutrition and post-operative infections was also shown [44].

## 5. Conclusions

1. Based on TNL value of the patients qualified for surgery malnutrition occurred for 46% of patients. After the procedure, the number of patients with malnutrition did not change significantly.

2. Based on BMI, patients were predominantly overweight (62.5%) and obese (26.0%). The BMI value after the surgery did not change significantly.

3. Based on MNA, 59% of patients before surgery were at risk of malnutrition. After surgery, the number of people at risk of malnutrition increased significantly by 50%.

4. Statistically, BMI values significantly affected the functional effectiveness of patients on the fifth postoperative day. The able-bodied patients more often had BMI in the normal range, whereas the patients with impaired functional status more often had BMI above the norm.

5. Complications after the surgery occurred in 35.4% of patients. A statistically significant BMI correlation was shown between complications occurring more frequently in people with underweight, obesity, and overweight than in people with normal BMI.

6. After coronary artery bypass surgery, patients have an abnormal state of nutrition which is the result of surgical trauma and is related to the limited self-reliance and the occurrence of postoperative complications. Therefore, it is important to apply a balanced diet set individually for each patient.

## Figures and Tables

**Table 1:** Statistical analysis of the nutritional status of patients who underwent CABG based on TNL.

Parameters	Research Time	X	SD	Me	Min	Max	*p*
n = 96 (100%)							
TNL	Before surgery	1862.59	2383.78	1525.00	1026.00	24613.00	0.0700
	One day after surgery	1566.62	550.90	1460.00	836.76	4876.00	
	Five days after surgery	1544.83	311.43	1501.50	846.00	3154.00	

TNL—total number of lymphocytes, X—average, SD—standard deviation, Me—median, Min—minimum, Max—Maksimum, *p*—statistical value.

**Table 2:** Nutritional status of patients based on TNL.

Parameters	Nutritional Status of Patients (n = 96)			*p*
TNL	Before	Proper	52 (54.2%)	-
		Mild malnutrition	37 (38.5%)	
		Moderate malnutrition	7 (7.3%)	
	First day	Proper	45 (46.9%)	0.1801
		Mild malnutrition	36 (37.5%)	
		Moderate malnutrition	15 (15.6%)	
	Fifth day	Proper	49 (51%)	0.9012
		Mild malnutrition	39 (40.6%)	
		Moderate malnutrition	8 (8.3%)	

TNL—total number of lymphocytes, *p*—statistical value.

**Table 3:** Statistical analysis of the nutritional status of patients who underwent CABG based on BMI.

Parameters	Research Time	X	SD	Me	Min	Max	*p*
n = 96 (100%)							
BMI	Before	27.92	2.65	28.07	18.25	33.51	0.4310
	Five days after	27.52	2.66	27.76	17.72	32.91	

X—average, SD—standard deviation, Me—median, Min—minimum, Max—Maksimum, *p*—statistical value.

**Table 4:** BMI of patients who underwent CABG before surgery and on the fifth day after the procedure.

BMI	Before (n = 96)	V day after (n = 96)
Underweight	2 (2.1%)	2 (2.1%)
Correct	9 (9.4%)	11 (11.5%)
Overweight	60 (62.5%)	67 (69.8%)
Obesity	25 (26.0%)	16 (16.7%)
*p*	-	0.4644

*p*—statistical value, BMI—body mass index.

**Table 5:** Statistical analysis of the nutritional status of patients who underwent CABG based on MNA parameter.

Parameters	Research Time	X	SD	Me	Min	Max	*p*
n = 96 (100%)							
MNA	Before	22.89	1.53	23	14	25	<0.0001
	Five days after	21.50	1.52	22	14	25	

X—mean, SD—standard deviation, Me—median, Min—minimum, Max—maximum, *p*—statistical value.

**Table 6:** Nutritional status of patients based on MNA.

Parameters	Nutritional Status (n = 96)			*p*
MNA	Before	Proper	38 (39.6%)	<0.0001
		Risk	57 (59.4%)	
		Malnutrition	1 (1%)	
	Five days after	Proper	5 (5.2%)	
		Risk	89 (92.7%)	
		Malnutrition	2 (2.1%)	

MNA—Minimal Nutritional Assessment, *p*—statistical value.

**Table 7:** Level of functionality of the patients before surgery and on the fifth day after surgery.

ADL		Before (n = 96)	V day after (n = 96)	*p*
Bathing	no	35 (36.5%)	95 (99.0%)	<0.0001
	yes	61 (63.5%)	1 (1.0%)	
Dressing	no	13 (13.5%)	49 (51.0%)	<0.0001
	yes	83 (86,5%)	47 (49.0%)	
Toilet hygiene	no	7 (7.3%)	32 (33.3%)	<0.0001
	yes	89 (92.7%)	64 (66.7%)	
Getting out of bed and into a chair	no	0 (0.0%)	3 (3.1%)	<0.0001
	yes	96 (100.0%)	93 (96.9%)	
Self-feeding	no	0 (0.0%)	0 (0.0%)	0.3666
	yes	96 (100.0%)	96 (100.0%)	
Self-control in urination and defecation	no	0 (0.0%)	1 (1.0%)	<0.0001
	yes	96 (100.0%)	95 (99.0%)	

ADL—Katza scale—Activities of Daily Living, *P*—statistical value.

**Table 8:** Relationship between the state of malnutrition (TNL) and the level of patients’ independent functional status (ADL).

Malnutrition	Impaired Functional Status	Proper Functional Status	*p*
TNL before CABG			
no	10 (55.6%)	42 (53.8%)	0.8956
yes	8 (44.4%)	36 (46.2%)	
TNL five days after CABG			
no	23 (35.9%)	26 (81.3%)	<0.0001
yes	41 (64.1%)	6 (18.8%)	

TNL—total number of lymphocytes, CABG—coronary artery bypass graftings, *p*—statistical value.

**Table 9:** Relationship between BMI and the level of the patients’ independent functioning (ADL).

BMI	Impaired Functional Status	Proper Functional Status	*p*
BMI before CABG			
underweight	0 (0.0%)	2 (2.6%)	<0.0001
normal	0 (0.0%)	9 (11.5%)	
overweight	2 (11.1%)	58 (74.4%)	
obesity	16 (88.9%)	9 (11.5%)	
	BMI fifth day after CABG		
underweight	2 (3.1%)	0 (0.0%)	0.0031
normal	2 (3.1%)	9 (28.1%)	
overweight	48 (75.0%)	19 (59.4%)	
obesity	12 (18.8%)	4 (12.5%)	

CABG—coronary artery bypass graftings, BMI—body mass index, *p*—statistical value.

**Table 10:** Relationship between nutritional status (MNA) and the level of patients’ self-functioning before surgery.

Nutritional Status	Impaired Functional Status	Proper Functional Status	*p*
MNA (before CABG)			
Correct	7 (38.9%)	31 (39.7%)	0.8848
Risk	11 (61.1%)	46 (59.0%)	
Malnourishment	0 (0.0%)	1 (1.3%)	
MNA (five days after CABG)			
Correct	3 (4.7%)	2 (6.3%)	0.5753
Risk	59 (92.2%)	30 (93.8%)	
Malnourishment	2 (3.1%)	0 (0.0%)	

MNA—Minimal Nutritional Assessment, *p*—statistical value.
